# Adequacy of Completion of Computed Tomography Scan Request Forms at a Tertiary Care Center in Pakistan: A Clinical Audit

**DOI:** 10.7759/cureus.3470

**Published:** 2018-10-20

**Authors:** Uffan Zafar, Aneesa Abid, Burza Ahmad, Hafiz Ali Ahmad, Fariha Zafar, Muhammad Usman Baig, Saba Akram

**Affiliations:** 1 Radiology, Bahawal Victoria Hospital, Quaid-E-Azam Medical College, Bahawalpur, PAK; 2 Psychiatry, Bahawal Victoria Hospital, Quaid-E-Azam Medical College, Bahawalpur, PAK; 3 Internal Medicine, Bahawal Victoria Hospital, Quaid-E-Azam Medical College, Bahawalpur, PAK; 4 Epidemiology and Public Health, Bahawal Victoria Hospital, Quaid-E-Azam Medical College, Bahawalpur, PAK

**Keywords:** request form, clinical audit, ct scan

## Abstract

Introduction

A lot of radiation exposure to a population comes from medical sources. Clinicians must justify the need for radiology procedures on a request form to prevent unnecessary scans and radiation exposure. Moreover, the properly-filled form will help to identify the patient correctly and provide clinical details to make a radiological diagnosis.

Objective

The purpose of the study was to audit the computed tomography (CT) scan request forms and find out the adequacy of completion of the request forms at Bahawal Victoria Hospital, Bahawalpur, Pakistan.

Materials and methods

We scrutinized 300 CT scan request forms received at the tertiary care center, Bahawal Victoria Hospital. We checked the adequacy of filling of different fields in the request forms like name, address, clinical and surgical history, and the name of required examination. We also looked for the missing subjects in the CT request form currently used in our hospital like the contact numbers of the patient and the doctor, the identity of the requesting doctor, renal function tests (RFTs), last menstrual period (LMP), and history of allergy. The results were analyzed using Statistical Package for the Social Sciences 20 (SPSS 20) (IBM, NY, USA) and Microsoft Office Excel Worksheet (Microsoft Corporation, NM, USA).

Results

The name of the patient was present in 100% of the request forms, surname in 27.66%, age in 73.33%, gender in 64.33%, date in 91.66%, bed number in 37.90%, address in 1% and the name of required examination in 99.6%. Information about diagnosis included clinical history in 50.66%, past surgical history in 1%, laboratory investigations in 1%, and clinical examination in 8.66% of the forms. All the forms had the name and signature of the referring consultant, but only 10.33% forms contained the identity of the requesting junior doctor. More than half of the request forms for CT scan provided RFTs. We found no record of the allergy, LMP, and the contact number of the doctor and the patient.

Conclusion

The information provided in the CT scan request forms was inadequate. The practice of filling these forms needs to be improved to protect the patients from unnecessary radiation exposure.

## Introduction

Worldwide, most of the radiation exposure comes from medical sources [1]. The collective radiation exposure from radiology to a population is thrice the exposure from nuclear power plants [2]. Much of this exposure is due to unnecessary or inappropriate scans which account for 20%-77% of total scans. So, the International Atomic Energy Agency (IAEA) has stressed a lot on the importance of justification of radiology scans [3]. We can considerably reduce this radiation exposure from medical sources if it becomes mandatory for clinicians to justify the need for a scan on a radiology request form. A clinician can do this by mentioning detailed clinical history, specific question, and history of previous radiation exposure on a radiology request form. It will help prevent unnecessary scans, thereby decreasing the radiation exposure.

The Royal College of Radiologists recommends filling the request forms completely [4]. The clinician should also mention the demographic details to recognize the patient correctly and to avoid mixing of reports. The patient’s contact number, ward and bed number are required to spot the patient if the radiologist needs the patient. The name, signature and contact number of the referring clinician should be mentioned [5]. A properly-filled request form is essential to understand the clinical problem and to make a radiological diagnosis [4]. If the radiologist requires any further information, he/she can contact the patient and the referring clinician by using the information on this form. So, it improves communication between radiology and other specialties. In this way it also prevents unnecessary delays in making reports.

Regular clinical audit enhances the quality of a health care system [6]. The European Society of Radiology recommends both internal and external audits in radiology [7]. The aim of this audit was to assess the adequacy of filling CT scan request forms in the radiology department. It will help to know about the degree of implementation of the principle of justification of the radiological procedures in Bahawal Victoria Hospital.

## Materials and methods

This was an audit that was conducted at Bahawal Victoria Hospital, a tertiary care center in Bahawalpur, Pakistan. We reviewed 300 computed tomography (CT) scan forms received in the radiology unit from May 16 to June 30, 2018. A data collection sheet was prepared and all the data was recorded in it. We checked the filling of demographic variables like name, surname, caste, age, gender, and date in the request forms. Other details that provided a justification of the scan and helped in making a correct radiological diagnosis, such as age, provisional diagnosis, brief clinical history, detailed history, past surgical history, physical examination, investigations, and the name of the requested radiology examination, were also assessed. The sheet also checked information about the name of the postgraduate trainee/medical officer/house officer, the referring consultant’s name and contact, the name of the ward or outpatient department, bed number, address, contact number, illegible handwriting, and non-standard forms. The CT scan request forms available in the hospital did not contain space for the patient’s contact number, history of allergy, renal function tests, last menstrual period of a woman of reproductive age, or previous investigations. These variables were also included in the sheet. Statistical Package for the Social Sciences 20 (SPSS 20) (IBM, NY, USA) and Microsoft Office Excel Worksheet (Microsoft Corporation, NM, USA) were used for data analysis.

## Results

**Figure 1:**
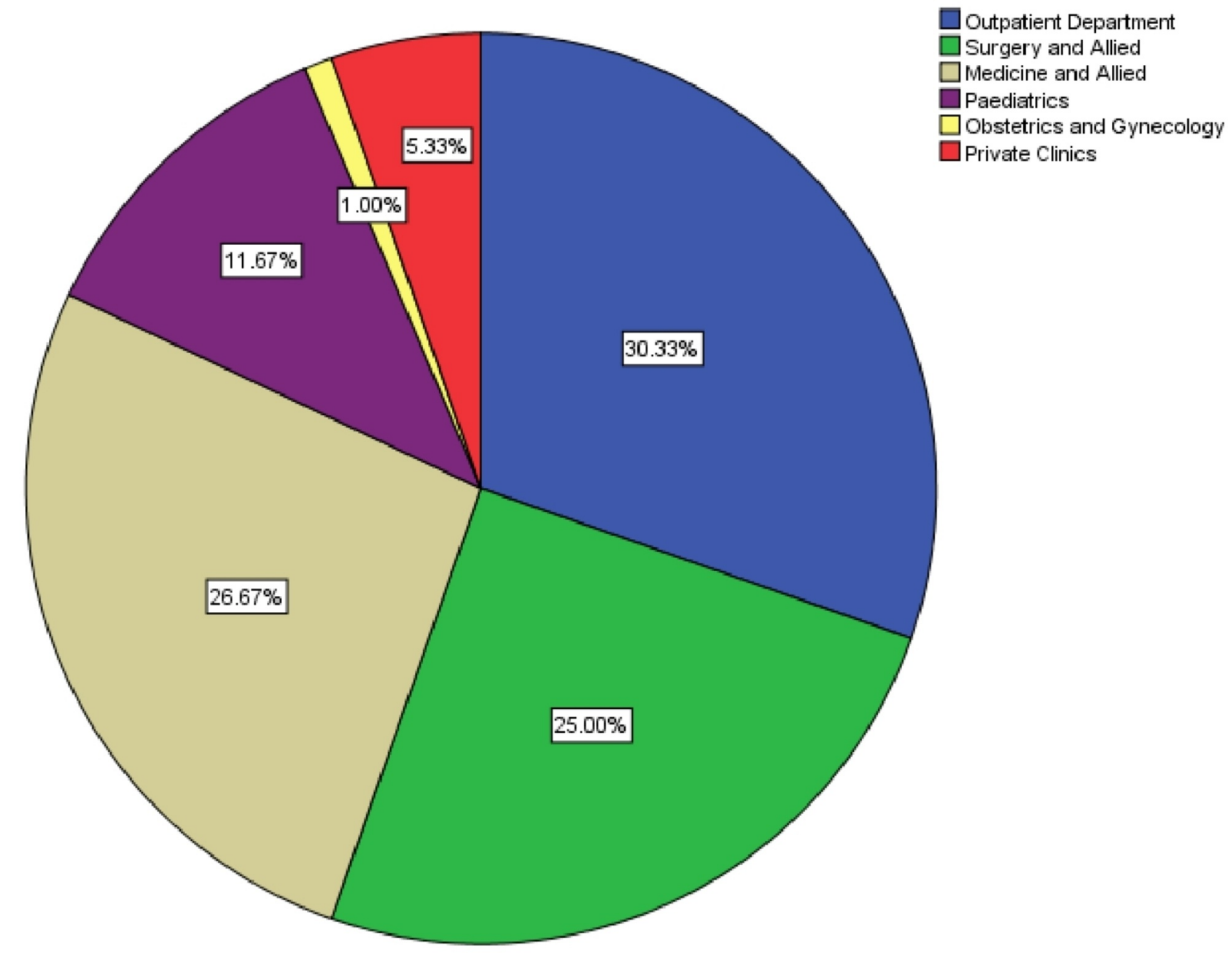
Source of the computed tomography (CT) scan request forms

Of the total 300 forms, no form was complete. We received nearly two-thirds of these 300 forms from different wards of the hospital and the remaining one-third from the outpatient department and private clinics. Figure 1 shows the percentage of sources of the CT scan request forms.

**Figure 2:**
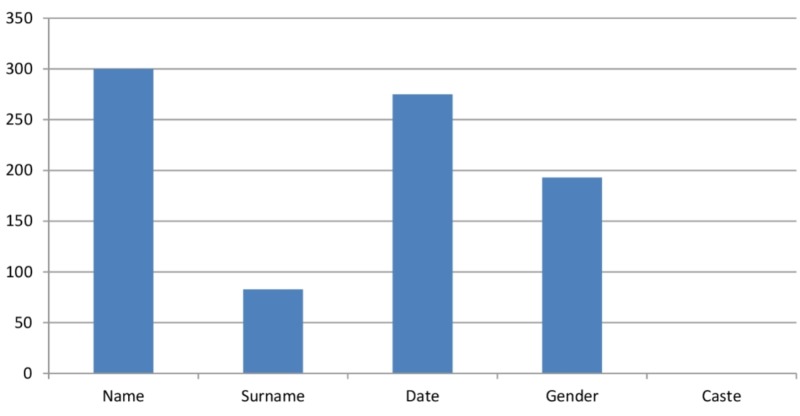
The frequency of provision of demographic variables in the computed tomography (CT) scan request forms

All the request forms mentioned the name of the patient, but the caste of the patient was missing. Surname and gender were not present in a large number of request forms. Figure 2 shows the frequency of providing the demographic details of patients in the CT scan request forms.

**Figure 3:**
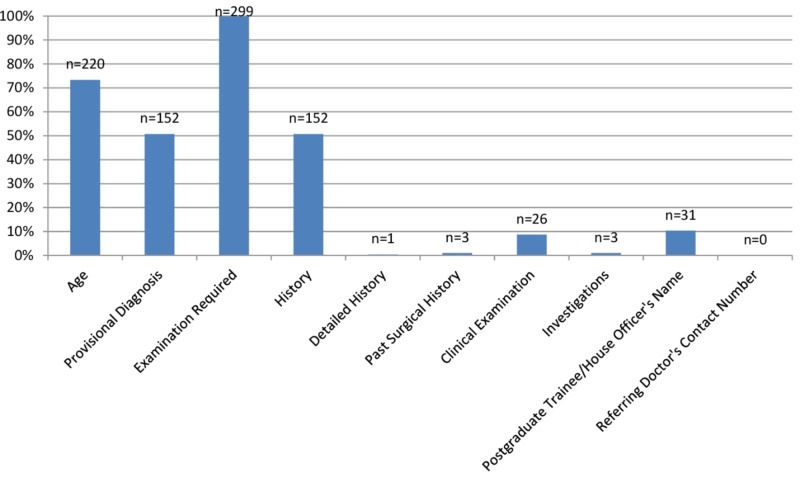
Provision of the details required to justify the need for a scan and to make a diagnosis

All the 208 request forms referred from inpatient departments contained the name of the ward. Only 79 (37.98%) of these had bed numbers. Just 1% of the total 300 forms mentioned the address of the patient. The specific question to be answered by the radiologists was present in only four request forms (1.33%). Other specialties did not ask for it. Only one form did not have the name of the required radiological examination in it. No form contained the contact number of any referring doctor. There was an omission of labs, past surgical history, and detailed history in 99% or more of the forms. Provisional diagnosis and brief history were absent in nearly half of the requests. Figure 3 provides the frequency of filling the details required to justify the need for a CT scan and to make a correct radiological diagnosis.

**Figure 4:**
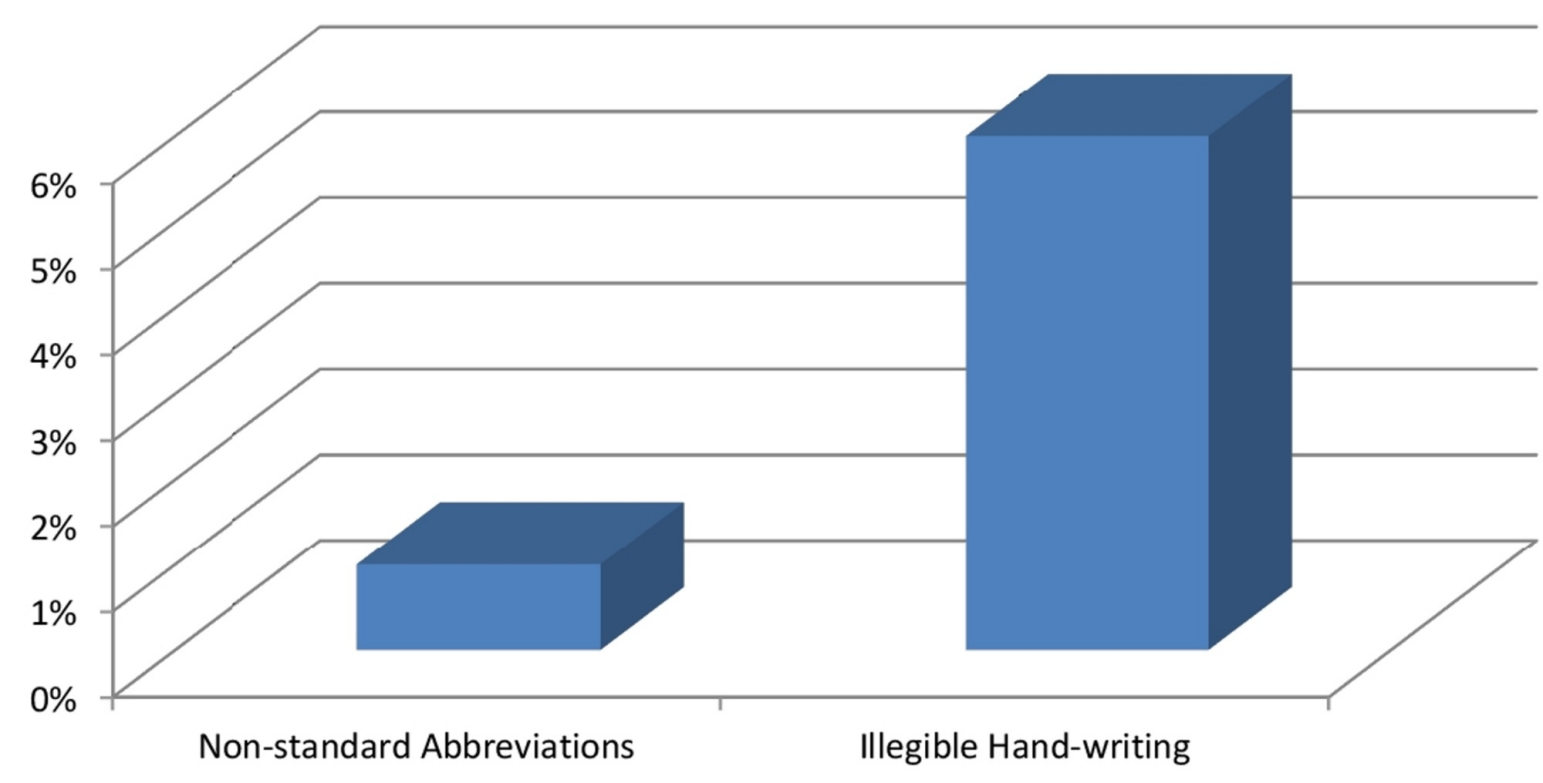
The percentage of the request forms with non-standard abbreviations and illegible handwriting

The history of allergy and last menstrual period (LMP) were not present. Renal function tests (RFTs) were missing in 27 out of the 58 forms of CT scan with intravenous (IV) contrast. All the request forms received from the outpatient department and private clinics were unconventional. These requests were on the prescription paper sheets. Figure 4 provides the percentage of the forms having non-standard abbreviations and illegible handwriting.

## Discussion

We found that nearly all of the CT scan request forms were incomplete. The information related to the identification of patients was not adequate. Lack of complete information to identify the patients can lead to mixing of their reports and thereby misdiagnosis and mismanagement. For the identification of a patient, we need the name, surname, caste, and date. In our study, the names of the patients were present in 100% of the forms. The two other studies show a percentage of 97.4% and 100% [8, 9]. We found surnames in 27.66% of the request forms. It was lesser compared to 100% surnames in another study [5]. We did not find the caste information of patients in any request form. A study conducted at three hospitals in Sudan showed that more than 90% forms mentioned the date [10]. While in our study, it was slightly better (91.66%).

CT request forms must reveal the ward's name, bed number and address of the patient for the delivery of reports. In our study, all the request forms (100%) had the name of the ward. It is better than the 77.3% and 98%, respectively, found in two other studies [9, 11]. Out of 208, only 79 (37.98%) forms had bed numbers and just 1% mentioned the address in our study. While in the two other studies 70% and 11.9% forms contained the address of the patient [11, 12].

Sometimes there is a need to contact the patient or the referring clinician to ask the questions related to the case. So, the request form must contain the name and contact number of the patient and the information about the referring clinician. Otherwise, there may be a delay in reporting the CT scan. We found that only 10.33% of the forms had the signatures of the postgraduate trainee or medical officer. While in another study, 15.6% forms had these signatures [5]. It is lesser as compared to another study in which 91% forms had both the names and signatures [13]. No form contained the contact number of the patient or attendant as there is no space for it in our CT request forms. While in two other studies, the percentages were 2.24% and 10%, respectively [8, 11].

Some diseases are common in a particular age group and gender. So, the radiologist requires these details of the patients to make a correct diagnosis. Moreover, it also helps to identify the patient correctly. In our study, the age was present in 73.33% of the forms, which is lesser than 90.3% and 83.45% in two other studies [5, 8]. We found that only 64.33% of the forms had the gender information. It is lesser than 96% that was found in a Nigerian study [12].

The radiologist should know about the medical and surgical history, laboratory investigations, provisional diagnosis, and the examination required to make a radiological diagnosis. Moreover, these details also help to justify the need for CT scans thereby preventing unnecessary CT scans and radiation exposure. The clinician must ask the specific questions for which he needs the help of the radiologist. Clinical history of the patient was present in 79.5% and 90%, respectively, in two studies [6, 14]. While in our study, it was comparatively lesser (50.66%). We found that only one form had a detailed history (0.33%) as compared to 18.5% in another study [5]. The past surgical history was present in only 1% of the forms, and it is lesser as compared to 59.8% but is comparable with 0.35% [8, 12]. In our study, 50.66% had a provisional diagnosis, and the investigation details were present in 1%. Another study had better percentages for provisional diagnosis (98%) and investigations (96%) [14]. Name of the examination required was present in 99.66% of the forms while it was 98.5% in another study [9]. We found that only four request forms (1.33%) asked specific questions and this percentage was far less as compared to 35% in another study [6].

A radiologist must ensure the safety of patients before performing the radiological procedures. So, the radiologist should know the history of allergy to some drug or intravenous (IV) contrast agent. The radiologist should have information about the recent renal function tests (RFTs) to decrease contrast-induced nephropathy risk and the last menstrual period to prevent fetal damage by ionizing radiations. For CT scan with IV contrast, 53.44% of the forms provided RFTs. This percentage is better than 1.5% in another study, but it is not adequate [6]. The radiology request forms available in Bahawal Victoria Hospital do not have space for the history of allergy and last menstrual period. So the request forms had no information about these fields in our study. In another study, 9% of the forms revealed the history of allergy [11]. Two studies mentioned LMP in 11.50% and 50.6% of the forms, respectively [8, 11].

A clinician and a radiologist can communicate via radiology request forms. Illegible handwriting, nonstandard abbreviations, and unconventional forms can affect this communication. It may result in misinterpretation, which can lead to a wrong examination, a repeat examination and thus more radiation exposure. Moreover, it may lead to a complete lack of understanding and thus cause a delay in the radiological examination. In our study, the handwriting was illegible in 6% of the forms, which is comparable with 8.6% and 7.37% in two studies [6, 8], respectively. We found nonstandard abbreviations in only 1% of the forms, while in another study these were present in 7.2% [6]. We received 30.33% of the CT scan request forms from the outpatient departments, and all of those were unconventional. These requests were on either white pages or simple prescription slips. Our percentage is nearly equal to another study in which 28% of the forms were unconventional [8].

We received 64.33% of the forms from the wards, 30.33% from the outpatient department, and 5.33% from private clinics. A study found that the requested scans were 64% from general medicine, 16% from surgery and 18% from obstetrics and gynecology departments [15]. While in our study we received 26.67% forms from medicine and allied, 25% from surgical and allied, 1% from obstetrics and gynecology, and 11.67% from pediatrics departments.

## Conclusions

Clinicians do not fill the radiology request forms adequately. They should justify the need for a scan to prevent unnecessary radiation exposure to patients. They should provide all the details that help in identifying the patient, ensuring patient safety, and making the diagnosis. There is a need to use standardized request forms that contain all the necessary details.
